# Disparate gain and loss of parasitic abilities among nematode lineages

**DOI:** 10.1371/journal.pone.0185445

**Published:** 2017-09-21

**Authors:** Martijn Holterman, Akbar Karegar, Paul Mooijman, Hanny van Megen, Sven van den Elsen, Mariette T. W. Vervoort, Casper W. Quist, Gerrit Karssen, Wilfrida Decraemer, Charles H. Opperman, David M. Bird, Jan Kammenga, Aska Goverse, Geert Smant, Johannes Helder

**Affiliations:** 1 Laboratory of Nematology, Department of Plant Sciences, Wageningen University, Wageningen, The Netherlands; 2 Department of Plant Protection, School of Agriculture, Shiraz University, Shiraz, Iran; 3 National Plant Protection Organization, Wageningen Nematode Collection, Wageningen, The Netherlands; 4 Nematology Research Unit, Department of Biology, Ghent University, Ghent, Belgium; 5 North Carolina State University, Department of Plant Pathology, Raleigh, United States of America; Institute of Botany, CHINA

## Abstract

Plant parasitism has arisen time and again in multiple phyla, including bacteria, fungi, insects and nematodes. In most of these organismal groups, the overwhelming diversity hampers a robust reconstruction of the origins and diversification patterns of this trophic lifestyle. Being a moderately diversified phylum with ≈ 4,100 plant parasites (15% of total biodiversity) subdivided over four independent lineages, nematodes constitute a major organismal group for which the genesis of plant parasitism could be mapped. Since substantial crop losses worldwide have been attributed to less than 1% of these plant parasites, research efforts are severely biased towards this minority. With the first molecular characterisation of numerous basal and supposedly harmless plant parasites as well as their non-parasitic relatives, we were able to generate a comprehensive molecular framework that allows for the reconstruction of trophic diversification for a complete phylum. In each lineage plant parasites reside in a single taxonomic grouping (family or order), and by taking the coverage of the next lower taxonomic level as a measure for representation, 50, 67, 100 and 85% of the known diversity was included. We revealed distinct gain and loss patterns with regard to plant parasitism *per se* as well as host exploitation strategies between these lineages. Our map of parasitic nematode biodiversity also revealed an unanticipated time reversal in which the two most ancient lineages showed the lowest level of ecological diversification and *vice versa*.

## Introduction

With insect herbivores as a major exception, most organismal groups from which lineages of plant pathogens and parasites would arise later on were already present in terrestrial habitats in Early Ordovician times (480 mya) when the first land plants evolved [1]. With 350,000 flowering plants species inhabiting terrestrial habitats [2], Angiosperms are the dominant food source of a remarkable diversity of herbivores, pathogens and parasites.

Nearly half of the 1 million documented insect species use plants as a food source [3]. Among the number of described fungal species (> 1.5 million), less than 10% (≈ 100,000) is capable of colonizing plants [4]. As compared to the previous groups, Oomycota, commonly referred to as water molds, show far less diversification (≈ 800 extant species), and within this class > 60% evolved a plant-parasitic lifestyle [5]. With about 27,000 described species, nematodes constitute a major group of mainly soil and sediment inhabitants from which about 15%, ≈ 4,100 species, use higher plants as their dominant food source [6]. All in all, the diversification of higher plants has resulted in an even greater diversification of plant attackers.

**Fig 1:**
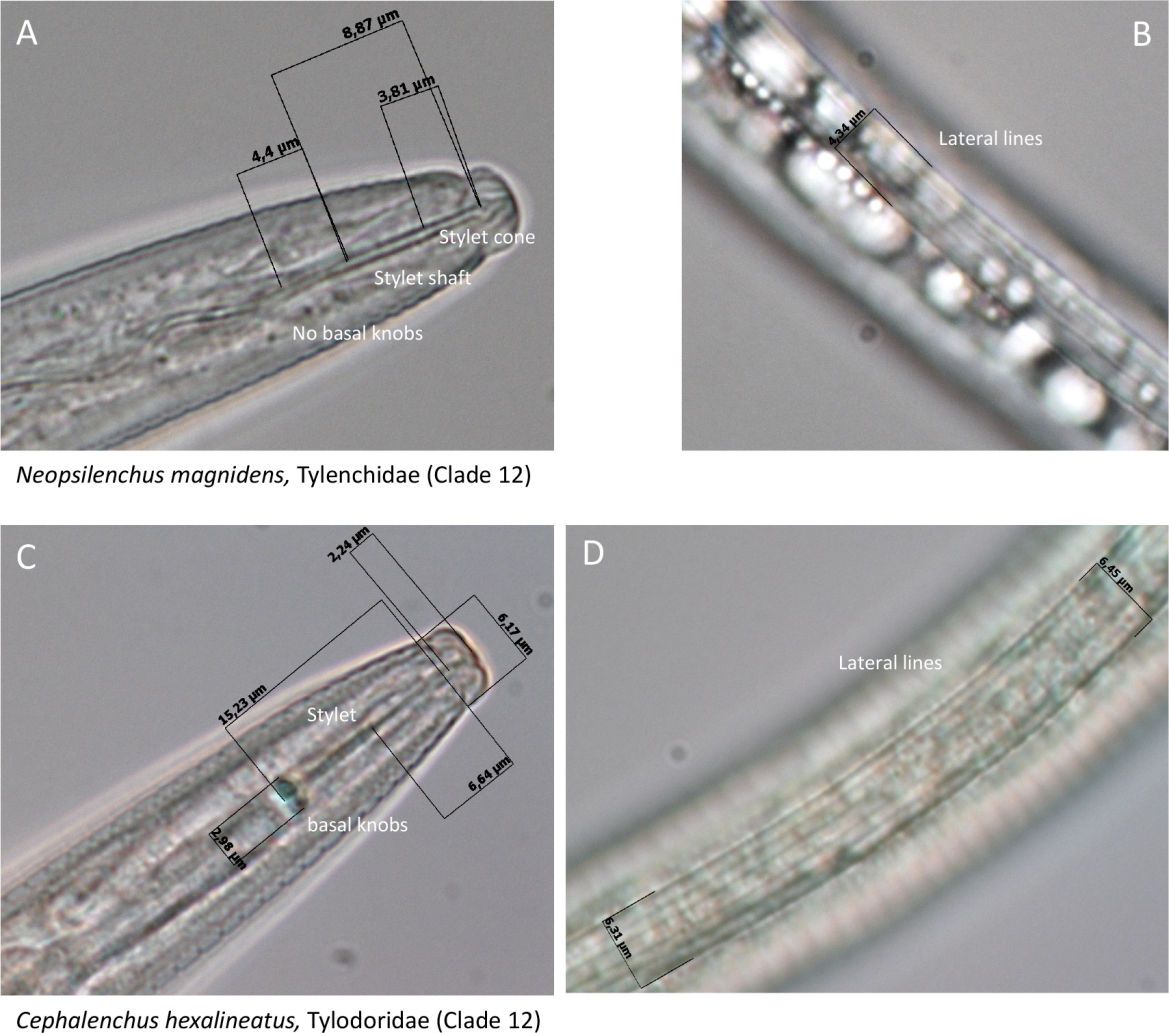
**Pictures of the head (A, C) and the middle regions (B, D) of two relatively basal representatives of the Tylenchida**. This speciose nematode order harbours most of the economically high impact plant-parasitic nematode species. Morphometrics of the stylet, an injection-needle like device used to puncture the plant cell wall (A, C), and the lateral field, indentations in the cuticle present in both sides of the nematode (B, D), are used for species identification. For these pictures, standard light microscopy was combined with differential interference contrast (DIC) optics (magnification: 1,000x).

**Fig 2:**
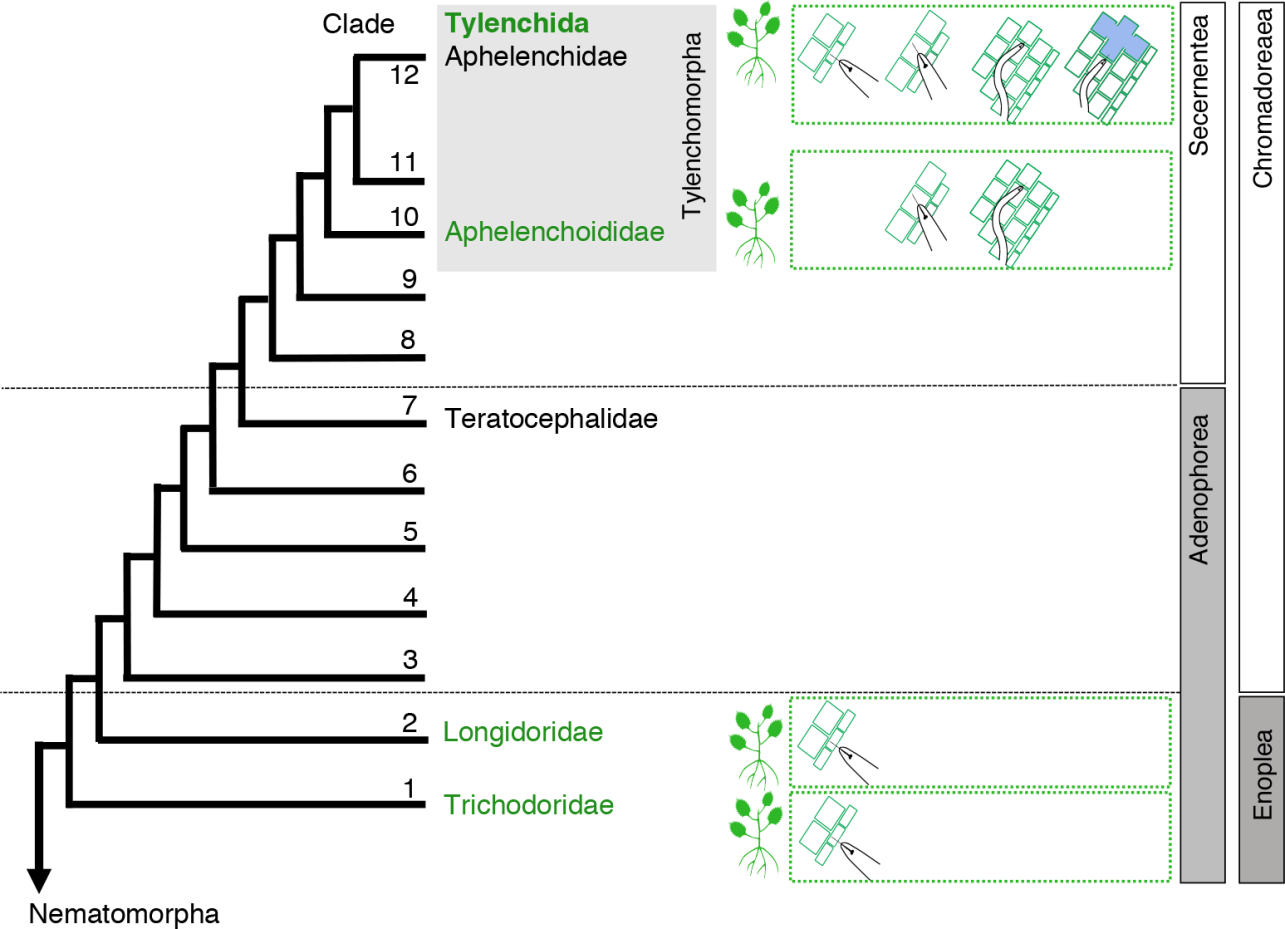
A generalized overview of the phylogenetic relationships within the phylum Nematoda based on (nearly) full-length small subunit ribosomal DNA (SSU rDNA) sequences. For clade designation, we adhered to Holterman et al. [7]. Plant parasites are found in Clades 1, 2, 10 and 12, and icons are used to distinguish four types of plant-parasitic nematodes: ectoparasites, semi-endoparasites, migratory endoparasites, and sedentary endoparasites.

Plant parasitism is a polyphyletic trait in most, if not all, organismal groups harboring representatives with this kind of trophic behavior. Among nematodes, four major lineages of plant-parasitic nematodes have been identified [7]. Plant-parasitic nematodes are equipped with a protrusible, injection needle-like device that is used to puncture the plant cell wall, to release effectors into the apoplast and inside plant cells, and (in most cases) to take up food from plant cells (Fig 1A and 1C). The morphology and ontogeny of these puncturing devices is lineage-specific. The presence of such a device is not an exclusive trait of plant parasites, predators such as members of the genera *Seinura* (Clade 10) and *Labronema* (Clade 2, for Clade overview see Fig 2) use it to puncture other nematodes, and feed on the body content [8].

Plant-parasitic nematodes are mainly below-ground parasites of higher plants, and they predominantly feed on plant roots. In most (agro-) ecosystems, the plant parasites constitute only a minority within the terrestrial nematode community; the majority of these assemblages feeds on bacteria, fungi and small eukaryotes such as protists. Although nematodes are typically present in high numbers (2–20 million per square meter), these vermiform organisms are non-obvious as they are colorless and relatively small with an average length of less than 1 mm. With an estimated capitalized damage of $US 118 billion per year (11% of production) [9], the economic impact of plant-parasitic nematodes is enormous. For major food crops such as soybean and potato cyst nematodes reside among the most serious yield-limiting factors. Tropical root-knot nematodes, a conglomerate of at least three highly polyphagous *Meloidogyne* species, are major pests in numerous vegetable crops and ornamentals throughout (sub)tropical regions of all continents [10].

A number of neutral, pathogenicity-unrelated markers have been explored to reveal evolutionary patterns within this speciose and trophically diverse animal phylum. Nematodes most likely arose in Early Cambrian, about 550 mya [11], and this implies that only highly conserved genes can be used for phylogenetic reconstruction. Phylum-wide studies published so far exploited the phylogenetic signals present in full-length small subunit ribosomal DNA (SSU rDNA) sequences [7, 12, 13]. Currently, 19 nematode genomes have been published and the sequences of dozens of genomes are soon to be released [14]. Although this will certainly change in the near future, the diversity of the current set is still limited with a strong bias towards nematode species with a high economical or health impact.

Nematodes harbor a limited number of informative morphological characters, and species identification requires ample expertise. Morphological expertise in invertebrate taxonomy is in decline worldwide, and this holds for nematology as well [15]. No DNA sequence information whatsoever was available for numerous basal (often harmless and ecologically barely characterized) plant-parasitic and fungivorous taxa. With a combination of morphological and molecular expertise we were able to close this knowledge gap to a substantial extent. Subsequently, we investigated whether the evolution of plant parasitism in four independent nematode branches resulted in similar or disparate diversification patterns with regard to (1) the genesis and loss of plant parasitism *per se* (once or multiple times), (2) the (in)ability to enter the host plant (ecto- *versus* endoparasitism), (3) preferences for below or aboveground parasitism (root *versus* stem and flower parasites), and (4) absence or presence of phoretic associations. A comprehensive molecular framework combined with two independent state-of-the-art phylogenetic algorithms allowed for the full exploitation of the phylogenetic signal present in full-length SSU rDNA sequences from ≈ 1,600 plant-parasitic nematode taxa and their close relatives. Comparison of the four lineages disclosed remarkably diverse gain and loss patterns with regard to the evolution of plant parasitism as well as the various manifestations thereof.

## Materials and methods

### Nematode collection and identification

Nematodes were collected from various soil habitats, and extracted using standard techniques (Oostenbrink 1960). Prior to DNA extraction, individual nematodes were identified using a light microscope (Zeiss Axioscope) equipped with differential interference contrast (DIC) optics. A CCD camera (AxioCam MRc5 (Zeiss)) was used to take a series of digital images from each nematode to retain the possibility to re-evaluate the identity of individual specimen. Series of digital images from individual nematodes are available upon request. For classification of plant-parasitic nematode taxa present Clades 1, 2 and 10 we adhered to Hunt [16, 17] and Decraemer and Geraert [18]. For the systematics of plant-parasitic taxa in Clade 12 we used the nomenclature proposed by Siddiqi [19].

### DNA extraction, amplification and sequencing of SSU rDNA

Total DNA was extracted and amplified from single nematodes. Individual nematodes were incubated in lysis buffer, and SSU rDNA (two overlapping fragments, in total spanning ≈ 1,700 bp) was amplified using three universal and one nematode-specific PCR primer as described by Holterman et al. [7]. Fragments were cloned in a TOPO TA pCR2.1 cloning vector, and sequenced using standard procedures.

### Sequence alignment ad phylogenetic analyses

Using the SSU rDNA sequence alignment as described in Holterman et al [7] as a guide line, ‘Fast aligner’ (one of the integrated aligner tools of ARB) was employed to integrate new sequences (newly generated and retrieved from GenBank) to the alignment. In case this tool was locally incapable to generate an acceptable alignment, secondary structure information as predicted by Mfold (http://unafold.rna.albany.edu/? q=mfold/RNA-Folding-Form) was used as guideline. Secondary structure information of *Loricera foveata* (Insecta, Carabidae) (http://bioinformatics.psb.ugent.be/webtools/rRNA/secmodel/index.html) was selected as the SSU rRNA secondary structure backbone. The secondary structure information, the definition of all individual loops and stems, was translated into a RAxML readable secondary structure file with home-made python scripts. For MrBayes, additional scripts were generated for the description of the pairs, the stem and loop charset.

Clades 1 and 2: RAxML analysis was performed on the CIPRES Science Gateway (RAxML on XSEDE 7.6) [20]. As secondary Structure Substitution models, GTR was used for the loop partitions, and S16A for the stem partitions. For MrBayes analyses (v3.2.3 x64), the doublet nucmodel was used for the stem partition, the 4by4 nucmodel for the loop partition. The temp setting was deliberately lowered from default setting 0.2 to 0.05 to get sufficient swaps between chains. Tracer v.1.6 [21] was used to confirm that parameters had converged.

Clades 10 and 12: For species that were represented by a large number of sequences, multiple consensus sequences were created to adequately capture the variation present in the species. For both MrBayes and RAxML analyses datasets were partitioned according to their secondary structure. RAxML-HPC2 was run with 1,000 bootstraps using the GTRCAT model (raxmlHPC-HYBRID -T 4 -n outfile -s infile.txt -x 12345 -N 1000 -q part.txt -k -c 25 -p 12345 -f a -m GTRCAT). Bayesian trees were created using the GTR + I + G model of nucleotide substitution and using 4 parallel runs with 4 chains each. The Clade 10 tree was run for 1 million generations and the Clade 12 tree was run for 10 million generations. The burnins were 100,000 and 500,000 generations respectively. Tracer v.1.6 [21] was used to confirm that parameters had converged.

## Results and discussion

### Minimizing sampling bias

Less than 1% of the ≈ 4,100 described plant-parasitic nematode species is well-studied as this minority is responsible for very significant losses in food and feed production worldwide [9, 10]. Sampling bias towards this minority of high-impact plant parasites may obscure evolutionary patterns on the origin and the diversification of this parasitic life style. Hence, we isolated and identified numerous, supposedly harmless plant parasites from so far non- or under-represented families and genera by their morphological and morphometric features, and further characterized these on the basis of their full-length SSU rDNA sequences. Fig 1 shows two examples of relatively basal plant-parasitic nematode species that can only be identified by an extensive set of morphometric characteristics.

The resulting data set comprised 1,673 full-length SSU rDNA sequences (Table 1). To assess the coverage per lineage, we compared the representation of taxa in our SSU rDNA framework with the relatively recent, authoritative systematic overviews. For plant-parasites in Clades 1, 2, and 10 we adhered to Hunt [16, 17], whereas for the highly speciose Clade 12 the systematics of Siddiqi [19] was followed. For three out of four clades, the coverage at relevant taxon level was ≥ 80% (Table 1), and it is concluded that the plant-parasitic nematode biodiversity is relatively well covered in our analyses.

**Table 1:** Taxon coverage for the four plant parasite-harboring nematode lineages.

Clade ID	Target taxon	♯ taxa described by Hunt [16, 17], Decraemer and Geraert [18], and Siddiqi [19]	Coverage	Ingroup: Number of SSU rDNA sequences	Outgroup Number of SSU rDNA sequences
**1**	Family Trichodoridae	6 genera (*)	50% (3 genera)**	93	13
**2**	Family Longidoridae	6 genera	67% (4 genera)	171	1
**10**	Family Aphelenchoididae	6 subfamilies	100% (6 subfamilies)	320	47
**12**	Order Tylenchida	27 families	85% (23 families)	1,089	10

* Unlike Hunt (1993), *Nanidorus* is considered here as a valid genus

** Relatively low coverage of the Trichodoridae because of the non-representation of three Neotropical genera, *Monotrichodorus*, *Allotrichodorus* and *Ecuadorus*.

### Positioning of plant-parasitic nematode lineages at phylum level

Within the phylum Nematoda, 12 clades have been defined on the basis of SSU rDNA sequences [7]. As shown in Fig 2, two major lineages of plant-parasitic nematodes reside in a basal position vis-à-vis Clade 7, whereas the two other lineages are positioned distally. This robustly supported Clade 7 harbours a single, monogeneric bacterivorous family comprising a single genus, *Teratocephalus*, and is considered as the immediate outgroup of all Secernentea [22] (Fig 2). Plant parasites in Clades 1 and 2 are all obligatory ectoparasites, each grouped within monophyletic families with limited diversification. Further diversification of parasites of higher plants took place in Clade 10 and, most explicitly, in Clade 12. Clade 10 comprises numerous fungivores and aboveground plant parasites, often vectored by insects. Clade 12 is in essence a highly variegated lineage of facultative and obligatory plant parasites. Clade 12 not only harbours the vast majority of all plant-parasitic nematode species, but can also be typified by the multitude of strategies to extract food from a wide range of plant tissues (Fig 2).

### Clades 1 and 2—two most basal, moderately diversified lineages exclusively harbour obligatory ectoparasites

**Fig 3:**
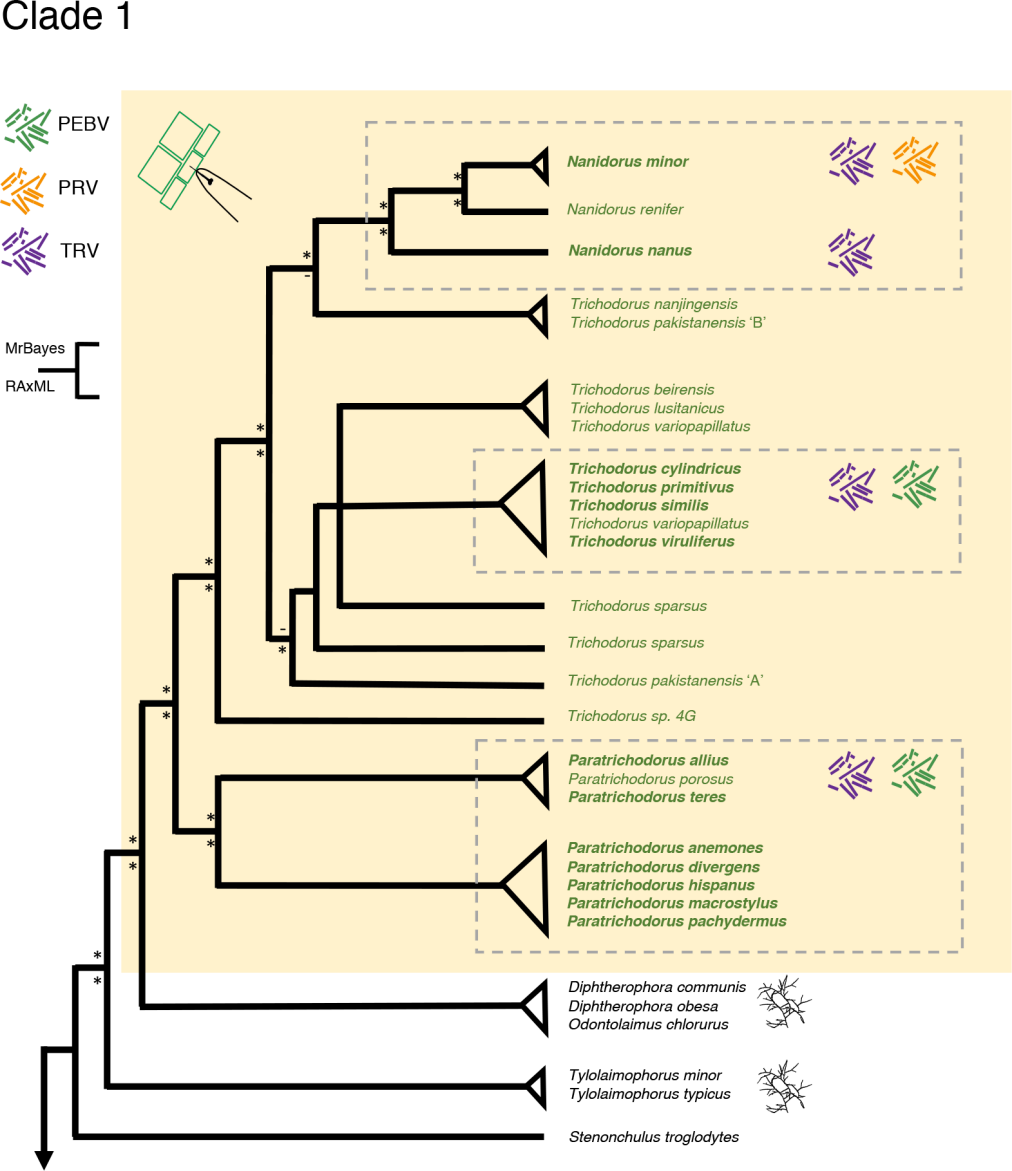
Simplified overview of the phylogenetic relationships within the family Trichoridae (Clade 1) based on (nearly) full-length SSU rDNA sequences. For full overview see S1 Fig. Symbols behind names represent specific association with plant viruses belonging to the genus *Tobravirus*. PEBV, pea early browning virus; PRV, pepper ringspot virus; TRV, tobacco rattle virus. Nematode species for which robust information about virus transmission could be found presented in bold. An asterisk near branching pointing refers to a posterior probability > 0.95, or a bootstrap value above 65%.

Clade 1 harbours a single lineage of obligatory plant parasites all belonging to a single family, Trichodoridae. With ≈110 described species [16, 23] this family is poorly diversified. Our analyses point at a sister relationship between the fungivorous genus *Diphtherophora* and the genus *Odontolaimus* on the one hand, and all members of the Trichodoridae on the other (Fig 3, S1 Fig). Both immediate outgroups of the Trichodoridae, the Diphtherophoridae and the Odontolaimidae are equipped with a dorsal tooth. In case of *Odontolaimus* this was described as a triangular elongated dorsal tooth. Hence, the ectoparasitic, obligatory members of the family Trichodoridae presumably arose from fungivorous ancestors equipped with a protrusible device allowing them to puncture enforced cell walls.

**Fig 4:**
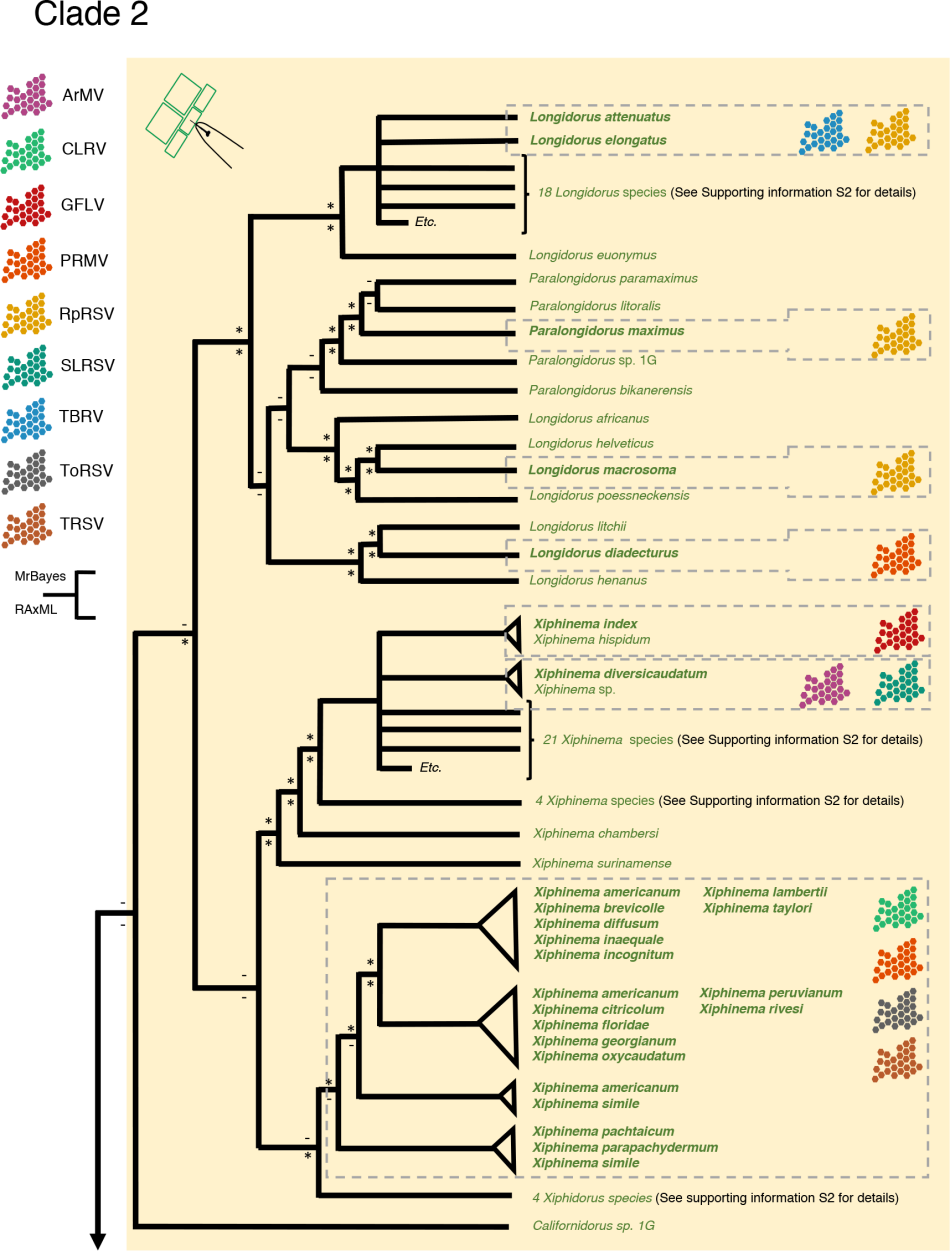
Simplified overview of the phylogenetic relationships within the family Longidoridae (Clade 2) based on (nearly) full-length SSU rDNA sequences. For full overview see S2 Fig. Symbols behind names represent specific association with plant viruses belonging to the genus *Nepovirus*. ArMV, Arabis mosaic virus; CRLV, cherry rasp leaf virus; GFLV, grapevine fanleaf virus; PRMV, peach rosette mosaic virus; RpRSV, raspberry ringspot virus; SLRSV, strawberry latent ringspot virus; TBRV, tomato black ring virus; ToRSV, tomato ringspot virus, TRSV, tobacco ringspot virus. Only for nematode species names in bold, robust information about virus transmission could be found. An asterisk near branching pointing refers to a posterior probability > 0.95, or a bootstrap value above 65%.

Unlike the positioning of the Trichodoridae in Clade 1, the exact position of the Longidoridae in Clade 2 is unclear. A single genus, *Californidorus*, is positioned sister to all Longidoridae, but this placement is not well supported. Its current, provisional placement at the very base of the Longidoridae is supported by morphological data [24]. The family Longidoridae is closely related to the Nordiidae, and *Californidorus* shows a mix of characters of both families [25]. The overall topology of the Longidoridae (Fig 4, S2 Fig) is largely congruent with a previous analysis based on the D2-D3 expansion regions of LSU rDNA [26]. It is concluded that the two basal lineages of plant-parasitic nematodes are each the result of two independent, single gain-of-function events.

### Multiple gains of virus transmission are exclusively observed in Clades 1 and 2

Some Trichodoridae transmit Tobraviruses. Our analyses show that the ability to act as a virus vector has arisen multiple times with this family. Within the *Trichodorus–Nanidorus* branch, virus transmission arose twice (Fig 3). One group that includes *T*. *primitivus*, *T*. *similis*, *T*. *cylindricus* and *T*. *viruliferus* fully corresponds to a set of *Trichodorus* that was grouped on the basis of composition of their body cuticle (referred to as ‘Type 1’ [27]). The second cluster consists of *Nanidorus minor* and *N*. *nanus* (occasionally the genus name ‘*Paratrichodorus’* is used for both species). The virus transmission status of the third species, *N*. *renifer*, is unknown [28]. *N*. *minor* is so far the only member of the Trichodoridae that was reported to transmit pepper ringspot virus (PepRSV). Most *Paratrichodorus* species are confirmed as vector of tobacco rattle virus (TRV) and/or pea early browning virus (PEBV) [29]. It is noted that virus transmission data for *Paratrichodorus divergens* and *P*. *porosus* are non-conclusive [30, 31], and no information is available on the vector status of *P*. *macrostylus*. As no contradicting data have been published so far, we hypothesize that all members of the genus *Paratrichodorus* have the ability to transmit plant viruses. Hence, superposition of transmission data [29] on the current Trichodoridae tree reveals three independent lineages (Fig 3).

Adhesion of Tobraviruses to the surface of the lumen wall and the surrounding oesophageal cavity requires the viral coat protein (CP) and a second viral protein named 2b, a non-structural protein. Current insights suggest the 2b protein forms a bridge between the nematode surface and the coat protein of the virus [32]. It should be noted that non-transmission could be brought about by lack of adhesion to the oesophageal lining, but also by a hampered release of the virus particle upon feeding of the nematode on a host plant [33]. Hence, we hypothesize the presumably commensalistic nematode-virus relationship is brought about by parallel subtle modifications of the surface characteristics of the oesophageal lining of the nematode. This gave rise to three independent lineages within the family Trichodoridae that (easily) bind and release Tobraviruses.

Just like the Trichodoridae, the family Longidoridae harbours a number of species that can act as vectors of plant viruses. As compared to the Trichodoridae, virus-transmitting Longidoridae species are more scattered over the phylogenetic tree, with *Xiphidorus* (and possibly the barely characterized genera *Australodorus* and *Paraxiphidorus)* being the only genus for which virus transmission has never been reported. A subset of the Longidoridae transmits Secoviridae such as Grapevine fanleaf virus (GFLV), Tomato black ring virus (TBRV) and Rasberry Ringspot (RpRSV) virus (Fig 4). Unlike the Trichodoridae, domains in the virus coat protein (CP) are directly responsible for adhesion to the odontostyle and/or oesophageal lining (without involvement of a 2b-like helper protein) [34]. Supposing that similar mechanisms underlie virus transmission by other Longidoridae, the scattered distribution of virus transmitting Longidoridae species suggests that relatively simple modifications in the cuticular lining of the mouthparts suffice to change of non-vector Longidorid species into a virus transmitter.

### Clade 10 –At least five independent transitions from fungivory to aboveground plant parasitism

**Fig 5:**
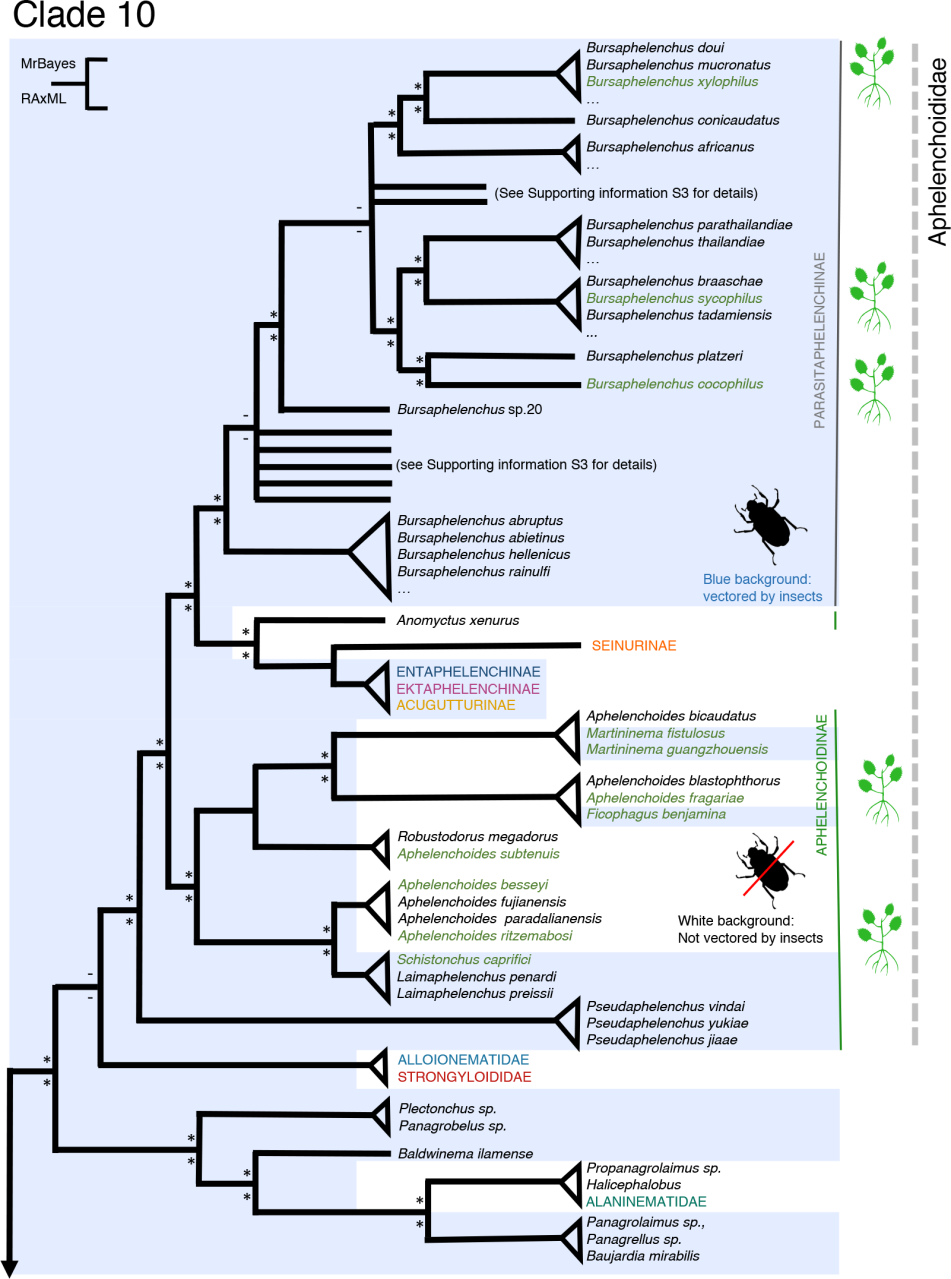
Simplified overview of the phylogenetic relationships within the family Aphelenchoididae and (Clade 10) based on (nearly) full-length SSU rDNA sequences. For full overview see S3 Fig. Plant-parasitic species are indicated in green, as well as by a plant icon in the right margin. Most non-plant-parasitic Aphelenchoididae are fungivores. A light blue background is used as an indicator for associations with insects. This may range from a simple phoretic interaction (*e*.*g*. *Bursaphelenchus sp*.) to obligate insect parasitism (*e*.*g*. *Entaphelenchus*). An asterisk near branching pointing refers to a posterior probability > 0.95, or a bootstrap value above 65%.

The remarkably scattered distribution of plant-parasitic nematode species within Clade 10 (Fig 5, S3 Fig) is most likely the result of convergent evolution. Within the subfamily Parasitaphelenchinae, the pine wood nematode *Bursaphelenchus xylophilus*, a facultative plant parasite that also feeds on fungi, arose independently from *B*. *cocophilus*, an obligatory plant parasite causing red ring disease in coconut and oil palm. On its turn, *B*. *sycophilus*, an obligate parasite of syconia (a syconium is a multiple fruits bearing inflorescence of figs) [35], evolved separately from the two afore-mentioned *Bursaphelenchus* species. It is noted that these three plant parasites depend on insect vectors for spreading and plant penetration. The main vectoring insects are respectively members of the insect genus *Monochamus* (Cerambycidae) [36], the palm weevil *Rhynchophorus palmarum* (Curculionidae) [37], and (presumably) the fig wasp species, *Ceratosolen appendiculatus* (Agaonidae) [35]. Hence, current phylogenetic as well as ecological data point at three independent transitions from fungivory to plant parasitism within the subfamily Parasitaphelenchinae.

Within the speciose genus *Aphelenchoides*, a predominantly fungivorous group within the subfamily Aphelenchoidinae, a few plant parasites evolved. Among these foliar nematodes, the causal agents of white tip in rice, *A*. *besseyi*, and the chrysanthemum foliar nematode *A*. *ritzemabosi* (both belonging to *Aphelenchoides* group 3[38]), arose independently from the strawberry crimp nematode *A*. *fragariae*, and *A*. *subtenuis* (group 2 [38]) (Fig 5). Within this subfamily, members of three other genera, *Schistonchus*, *Ficophagus* and *Martininema* [39], are resident. They specifically feed on fig inflorescences (‘syconia’). The presence of hypertrophied and damaged cells suggest that they feed on plant tissue, but the presence of yeasts (Saccharomycotina) in these syconia [40] might point at an alternative food source. However, it should be noted that attempts to culture *Ficophagus laevigatus* on medium with yeasts or fungi present in syconia failed [41]. Poor resolution at some nodes in combination with the scarcity of accurate information on feeding behaviour among the genera *Schistonchus*, *Ficophagus* and *Martininema*, prompted us to conservatively assess the number of transition events towards plant parasitism among the Aphelenchina at two.

### Clade 10 is characterised by repeated loss and secondary gain of insect associations

The current, relatively comprehensive phylogenetic analyses of Clade 10 suggest that fungivory and phoretic association with insects are ancestral character states. The most basal subclade of this Aphelenchoidinae—Parasitaphelenchinae dominated clade is defined by mycophagous members of the genus *Pseudaphelenchus* (Fig 5). The former is phoretically associated with subterranean termites [42], whereas the members of the latter genus are usually found in association with wood-boring insects [16, 43]. Although it received moderate support only (BI, pp 0.98, ML bootstrap value 69%), the sister relationship between the Aphelenchoididae and the Panagrolaimidae confirms the phoretic association with insects as an ancestral state; basal representatives of the family Panagrolaimidae are bacterivores vectored by insects (Fig 5).

All members of the Parasitaphelenchinae are associated with beetles, most of them with bark beetles (Scolytidae). In our analyses, the Aphelenchoidinae appear as a poly- and paraphyletic group, within which several loss and gain events could be pinpointed with regard to their phoretic association with insects (Fig 5). Most members of the genus *Aphelenchoides*, including the four plant-parasitic species, have lost their association with insects. The current analyses point at independent losses of insect association for the two sets of plant-parasitic *Aphelenchoides* species, *A*. *besseyi* and *A*. *ritzemabosi* on the one hand, *A*. *fragariae* and *A*. *subtenuis* on the other. Our analyses show two independent secondary gains of phoretic association with insects. Both fig-associated genera *Ficophagus* and *Martininema* are internally phoretic being carried in the haemolymph of abdomen of fig wasps, but members of these genera don’t show exclusive relationships with specific Agaonidae species [39].

### Clade 12 –the most successful lineage of plant parasites is characterized by a single major transition towards plant parasitism, followed by a loss and secondary gain event

**Fig 6:**
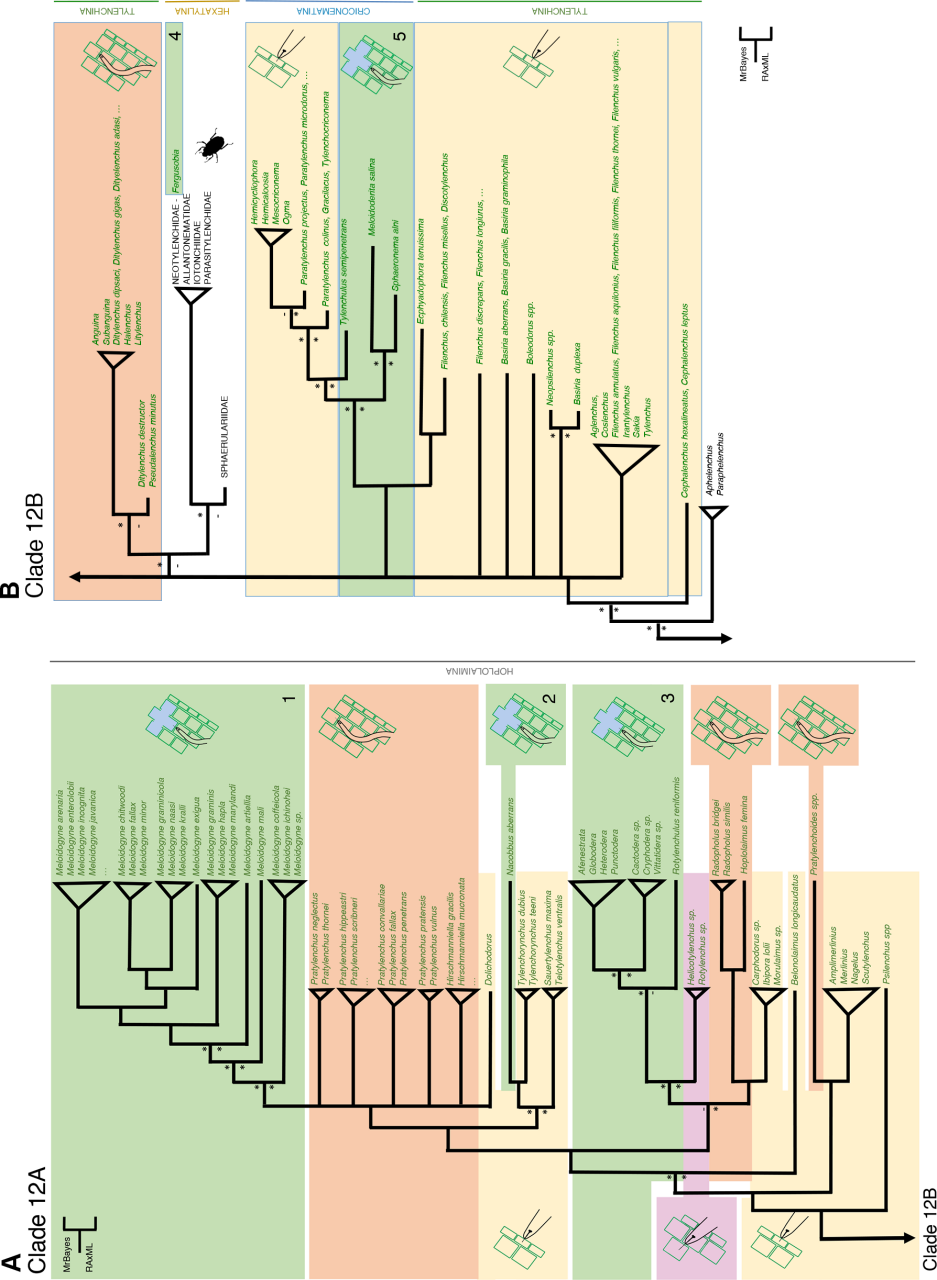
**A, B**. **Simplified overview of the phylogenetic relationships within Clade 12 (suborder Hoplolaimina, order Tylenchida) based on (nearly) full-length SSU rDNA sequences**. For full overview see S4 Fig. An asterisk near branching pointing refers to a posterior probability > 0.95, or a bootstrap value above 65%.

Analyses of 1,089 (nearly) full length SSU rDNA sequences covering 85% of the described Tylenchida families robustly supports the positioning of the predominantly fungivorous family Aphelenchidae as the immediate outgroup to all Tylenchida, an order that harbours virtually all economically high impact plant-parasitic nematodes. This positioning has been hypothesized before [7, 44], but only with newly generated molecular data from dozens of representatives of the most basal Tylenchida families were we able generate data to properly pinpoint this localisation (Fig 6A).

Our molecular framework constitutes support for three out of the four suborders together constituting the highly diversified order Tylenchida [19]. Two of these well-supported suborders (Hoplolaimina and Criconematina) harbour exclusively obligatory parasites of vascular plants, whereas the third one, Hexatylina, consists of predominantly of insect parasites. Tylenchina, the fourth and most basal suborder, appears as a poly- and paraphyletic group in our analyses. Members of the Tylenchina are trophically diverse as they may feed on fungi and lichen, on lower plants (algae and mosses), and on higher plants as ectoparasites (Fig 6A and 6B).

The primary entomopathogenic suborder Hexatylina constitutes a single monophyletic group that realized a major host shift from plant to insect parasitism (often alternated with a mycetophagous life stage). On the basis of shared morphological and biological characteristics, it was presumed to be closely related to the Anguinidae [19], a family of fungivores and parasites of aboveground plant parts, but a sister positioning with this family was only supported by BI. In a most distal branch of the Hexatylina, in general characterized by loss of plant parasitism, a single genus, *Fergusobia*, evolved a dicyclic life cycle alternating an insect-parasitic with a plant-parasitic generation. As such, *Fergusobia* constitutes the first example of secondary gain of plant parasitism within the phylum Nematoda.

### Clade 12 –Five evolutionary pathways leading to sedentary endoparasitism of plants

The current, relatively versatile analysis of the highly diversified Clade 12 revealed five independent and distinct evolutionary pathways leading to sedentary endoparasitism (Fig 6A and 6B, S4 Fig). Among plant-parasitic nematodes, sedentary endoparasitism is characterized by non-mobile, swollen females repeatedly feeding on limited group of re-differentiated plant cells. Below, we will pinpoint and discuss five independent origins of sedentary endoparasitism:

1. Root-knot nematodes (Meloidogynidae) (Fig 6A) arose from migratory endoparasites belonging to the Pratylenchidae subfamilies Pratylenchinae or Hirschmanniellinae. Large multinucleate “giant cells” are induced by second-stage juveniles (J2), and exploited as a sole food source throughout the life cycle of root-knot nematodes
2. The false root-knot nematode *Nacobbus aberrans* was nested within a family of ectoparasitic root surface feeders, the Telotylenchidae (Fig 6A), an unexpected positioning as this nematode is known as a member of the Pratylenchidae. Together with *Nacobbus*, this family was positioned with reasonable support at the base of a major branch harbouring the lesion and root-knot nematodes. In case of the false root-knot nematode, J2, J3 and J4 juvenile stages migrate in and out the root of a host plant, and only adult females induce the formation of a feeding site. The multinucleate nature of the resulting syncytium is not the result of induced nuclear divisions (as in case of root-knot nematodes), but it is the outcome of protoplast fusions between neighbouring cells. In this aspect feeding cells of false root-knot nematodes resemble syncytia induced by cyst nematodes (Heteroderidae).
3. Our analyses point at a common ancestry of the mainly temperate climate zone-bound sedentary endoparasitic cyst nematodes (Heteroderidae) and predominantly (sub)tropical reniform nematodes (Rotylenchulidae) (Fig 6A). A sister relationship between the Heteroderidae and the semi-endoparasitic Rotylenchulidae was supported by BI only. However, recent effector studies support the relatedness of reniform and cyst nematodes. CLAVATA3/ESR-s (CLE) are peptide hormones in plants, and mimics thereof, nematode-produced and secreted CLE-like proteins, are involved in syncytium formation [45]. These proteins show high similarity to homologues protein in cyst nematodes, and are only distantly related to CLE peptides from root-knot nematodes. Similarly, a GH5 endoglucanase from *R*. *reniformis* was shown to be most similar to an equivalent cellulase from the soybean cyst nematode *Heterodera glycines* (Hg-ENG-6) [46]. Hence, comparative effector studies constitute support for the relatedness between temperate cyst and tropical reniform nematodes.
4. A remarkably sudden switch towards sedentary endoparasitism is observed in the suborder Hexatylina, monophyletic branch which representatives typically alternate between (primarily) insect-parasitic and mycetophagous life stages (Fig 6B). Within this suborder, sedentary endoparasitism arose in a single, distally positioned genus, *Fergusobia*. Nematodes of this genus have a mutualistic relationship with *Fergusonina*, a genus of true flies found in Australasia only. *Fergusonina* flies in association with specific *Fergusobia* species induce galls in Myrtaceae (for recent review see [47]). In this mutualistic relationship, the nematode is vectored by the fly, and—upon deposition on a proper host plant—pharyngeal gland secretions of the nematode are at least co-responsible for gall formation [48]. Before the onset of feeding, *Fergusobia* juveniles induce the formation of hypertrophied, uninucleate plant cells. It should be noted that the multiple layers hypertrophied cells inside the galls do not resemble syncytia or giant cells as described above. Shortly thereafter, the female becomes semi-obese. Among nematodes, this is so far the only example of sedentary endoparasitism arising directly from insect parasitism.
5. At the base of the suborder Criconematina, two well-supported, distinct branches are observed that harbour representatives of the sedentary endoparasitic families Sphaeronematidae, and—in a next well-supported branch–Tylenchulidae (Fig 6B). The remarkable basal positioning of these sedentary endoparasites within the Criconematina is supported by the presence of sensory organs, phasmids, in the tail regions of the members of these two families [49]. In all other members of this suborder phasmids are absent, and this phenomenon should be considered as a result of secondary loss [19]. *Sphaeronema* juveniles (J2, J3, J4) feed ectoparasitically, and females induce a syncytium within the vascular cylinder. Syncytia induced by *Sphaeronema alni* females on chestnut secondary roots were characterized as connected mono-nucleate cells with dense cytoplasm and enlarged nuclei and nucleoli [50]. Unlike *Sphaeronema* juveniles, hatched J2s of *Tylenchulus semipenetrans* do not feed, and develop with a few days into adults. In the cortex, adult females induce the formation of multiple, hypertrophied ‘nurse cells’ with enlarged nuclei in the cortex. Unlike syncytia, there is no cytoplasmic continuity between the nurse cells, connectivity is facilitated by numerous plasmodesmata and nematode-induced feeding tubes [51]. The general view of sedentary endoparasitism as being the most evolutionary derived form of plant parasitism is questioned by these results.

By definition sedentary endoparasitism coincides with feeding site formation. Phylogenetic analyses based on SSU rDNA, a gene unrelated to pathogenicity, pinpointed five evolutionary pathways resulting in a form of sedentary endoparasitism. A closer look at pathogenicity-related biological characteristics revealed essential differences supporting the separate origin of these five lineages:

Feeding site induction maybe induced by parasitic J2s (1, 4), by adult females (2, 5), or by a mix of both approaches in a single lineage (3; parasitic J2s for cyst nematodes, adult females for reniform nematodes). Per lineage, feeding sites may be multinucleate (1) because of karyokinesis without cytokinesis, or (2, 3) due to cell fusion without karyokinesis, mono-nucleate (4), or a mix of either multiple, mono-nucleate hypertrophied cells (5, *Tylenchulus* spp.) or a syncytium (5, *Sphaeronema* spp.). Moreover, sedentary endoparasites may arise from migratory endoparasitic plant parasites (1, 3), from ectoparasites (2, 5), and even directly from insect parasites occasionally feeding on fungi (4).

## Conclusions

Detailed investigation of the origins of plant parasitism within and among four major nematode clades revealed remarkable differences in ecological diversification between the individual lineages. Whereas the two most basal and ancient lineages (Clades 1 and 2) are characterized by single transitions towards plant parasitism, plant parasitism arose at least five times in Clade 10. The most distal Clade 12, characterized by an enormous diversification of plant parasitic strategies, showed a major loss event and secondary gain of this trophic ability in the suborder Hexatylina. Moreover, Clade 10, a branch dominated by aboveground fungivores and plant parasites stands out in the frequent phoretic relationships with insects. Here, the absence of a relationship with insects should be regarded as secondary loss. The most distal and by far most diversified lineage, Clade 12, is signalized by a series of gradual transitions from fungivores, via facultative plant parasites feeding as well on algae and mosses towards obligatory plant parasites. Hence, the current diversity of plant-parasitic nematodes should be seen as the result of surprisingly disparate diversification processes branching out from four independent lineages.

## Supporting information

S1 Fig SSU rDNA-based phylogeny of Trichodoridae (BI and RAxML). (PDF)

S2 Fig SSU rDNA-based phylogeny of Longidoridae (BI and RAxML). (PDF)

S3 Fig SSU rDNA-based phylogeny of Aphelenchoididae (BI and RAxML). (PDF)

S4 Fig SSU rDNA-based phylogeny of Tylenchida (BI and RAxML). (PDF)

S1 File Taxon names and GenBank accession number per major lineage. (XLSX)
