# Supplementary figures and images for: Disparate gain and loss of parasitic abilities among nematode lineages
Source: PLoS One. 2017 Sep 21;12(9):e0185445. doi: 10.1371/journal.pone.0185445 (PMC5608397; doi:10.1371/journal.pone.0185445)

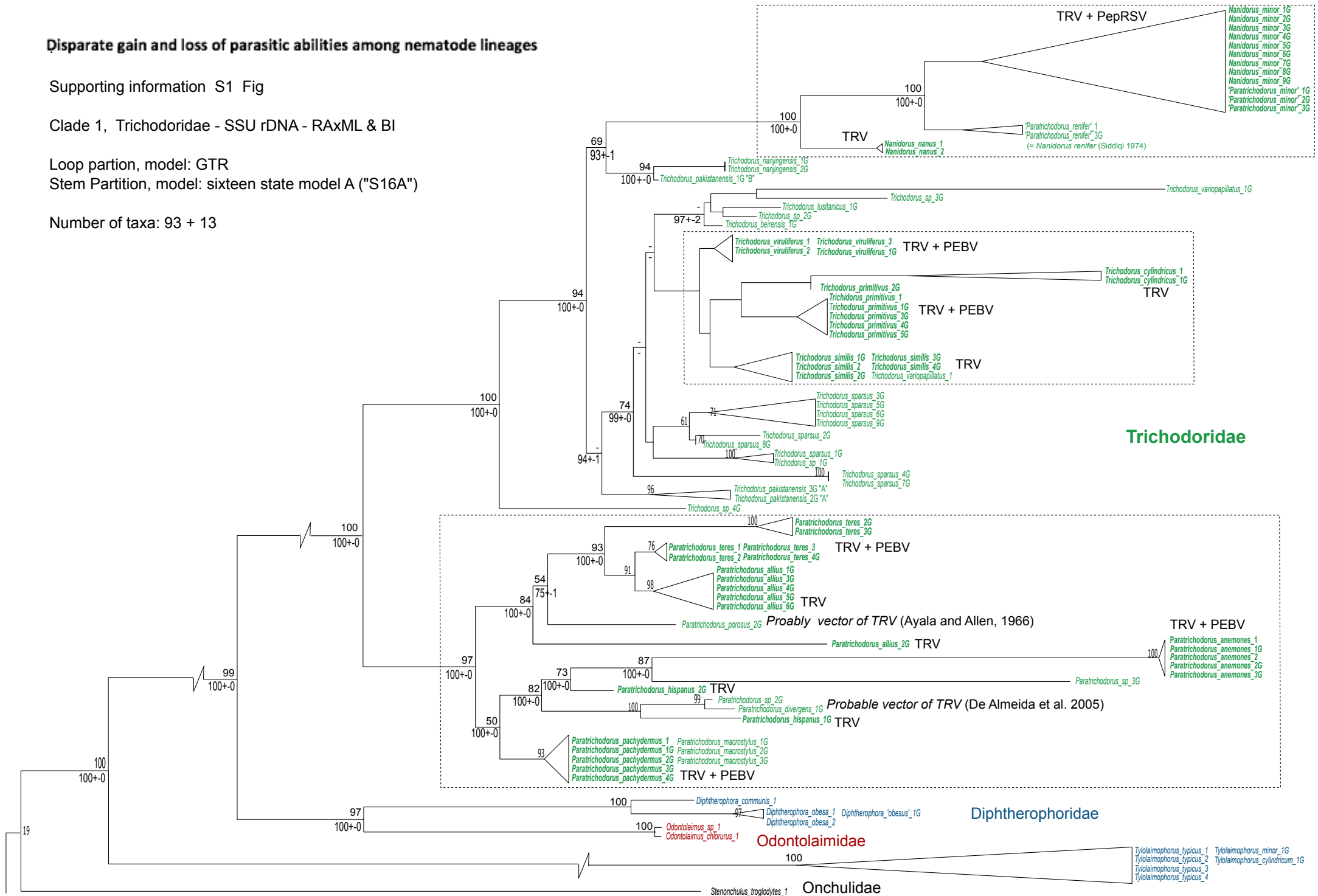

Supplement: S1 Fig — (PDF) [file pone.0185445.s001.pdf]

Aphelenchoididae

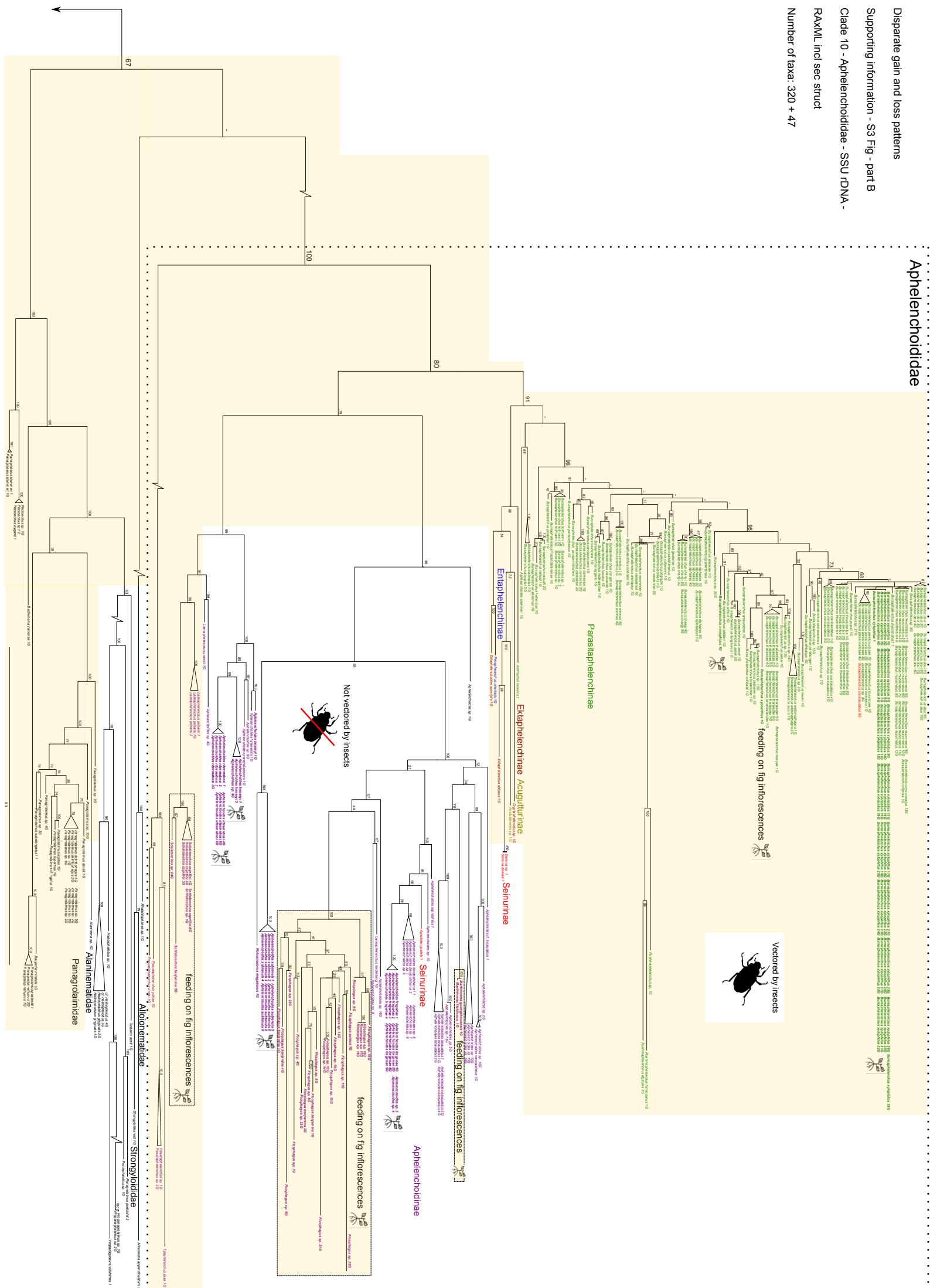

Supplement: S3 Fig — (PDF) [file pone.0185445.s003.pdf]
