# Supplementary material for: Disparate gain and loss of parasitic abilities among nematode lineages
Source: PLoS One. 2017 Sep 21;12(9):e0185445. doi: 10.1371/journal.pone.0185445 (PMC5608397; doi:10.1371/journal.pone.0185445)

Disparate gain and loss patterns

Supporting information S2 Fig - part A

Clade 2, Longidoridae - SSU rDNA - BI

70,000,000 generations
Average Deviation of split frequencies 0.009364 (= below 0.01)

Number of taxa: 171 + 1

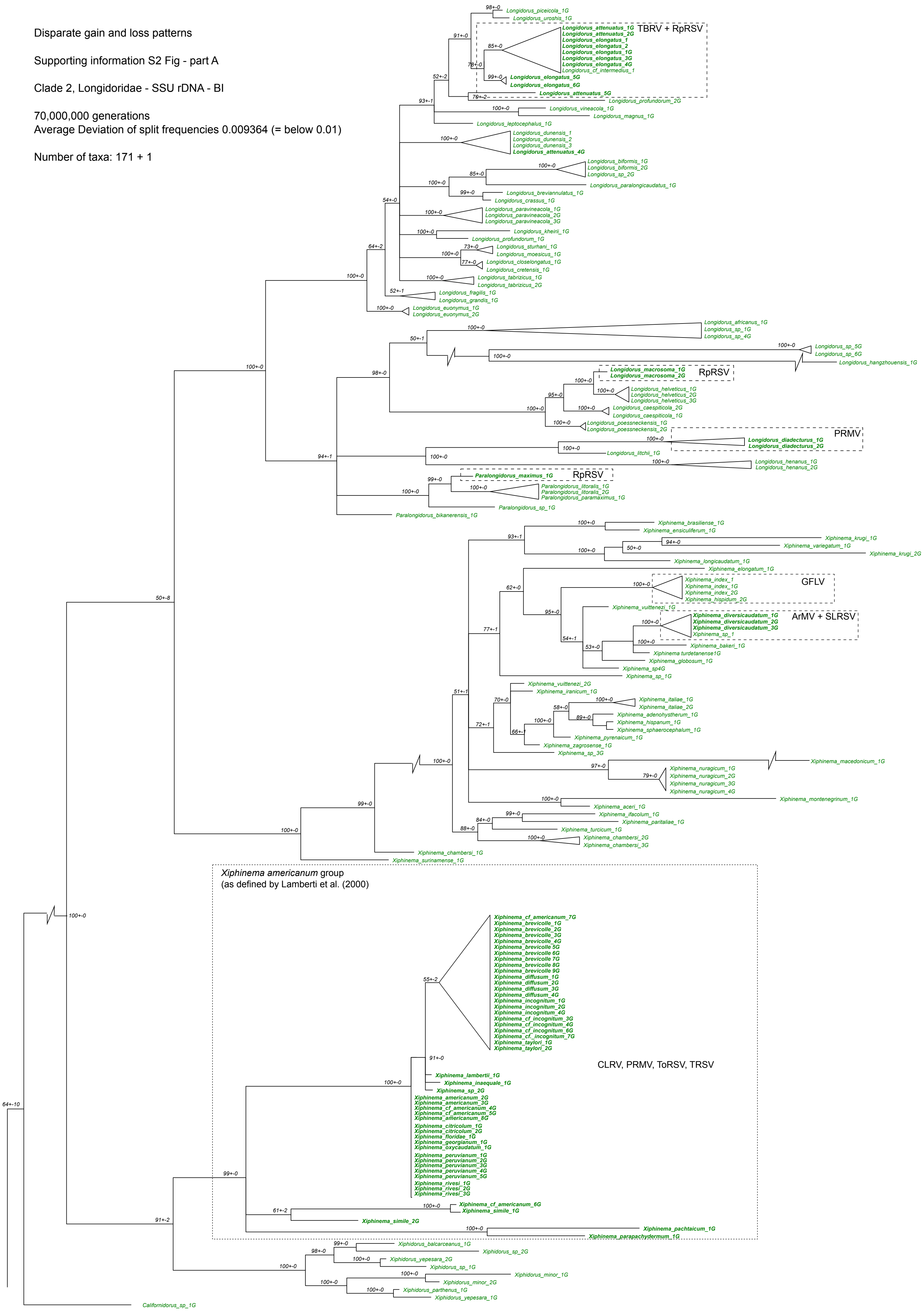

Disparate gain and loss patterns

Supporting information - S2 Fig - part B

Clade 2, Longodoridae - SSU rDNA - RAxML on XSEDE 7.6.3.
Loop partition: GTR
Stem partition: S16A

Number of taxa: 171 + 1

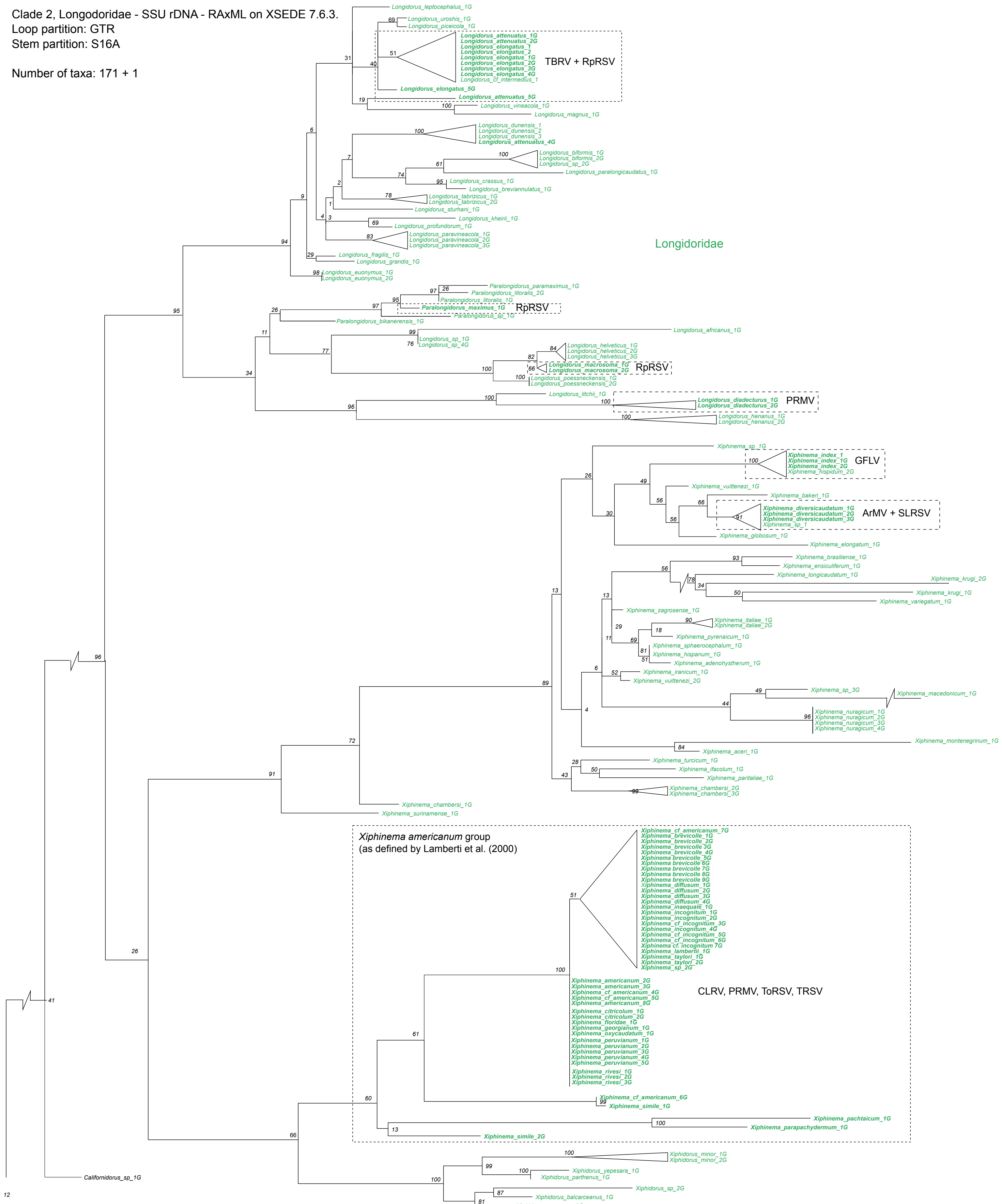

Supplement: S2 Fig — (PDF) [file pone.0185445.s002.pdf]
