# Supplementary material for: Disparate gain and loss of parasitic abilities among nematode lineages
Source: PLoS One. 2017 Sep 21;12(9):e0185445. doi: 10.1371/journal.pone.0185445 (PMC5608397; doi:10.1371/journal.pone.0185445)

Disparate gain ad loss patterns

Supporting information - S4 Fig - part A

Clade 12, Tylenchida - SSU rDNA

Bayesian inference incl sec struct

10,000,000 generations

Number of taxa: 1,089 + 10

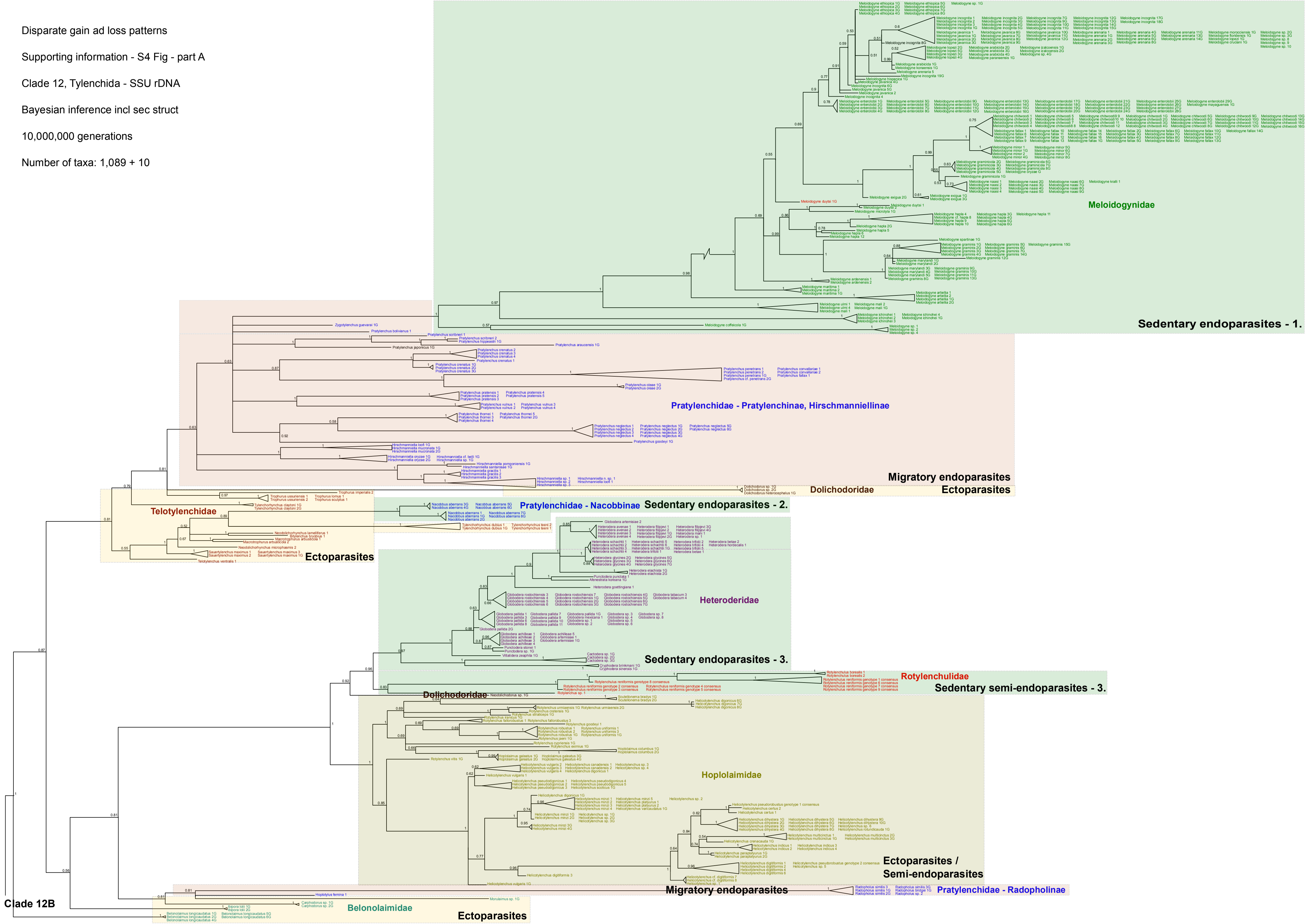

Holterman, Karegar and Mooijman et al.

Supporting Information - S4 Fig - part B

Clade 12, Tylenchida - SSU rDNA

RAxML incl sec struct

Number of taxa: 1,089 + 10

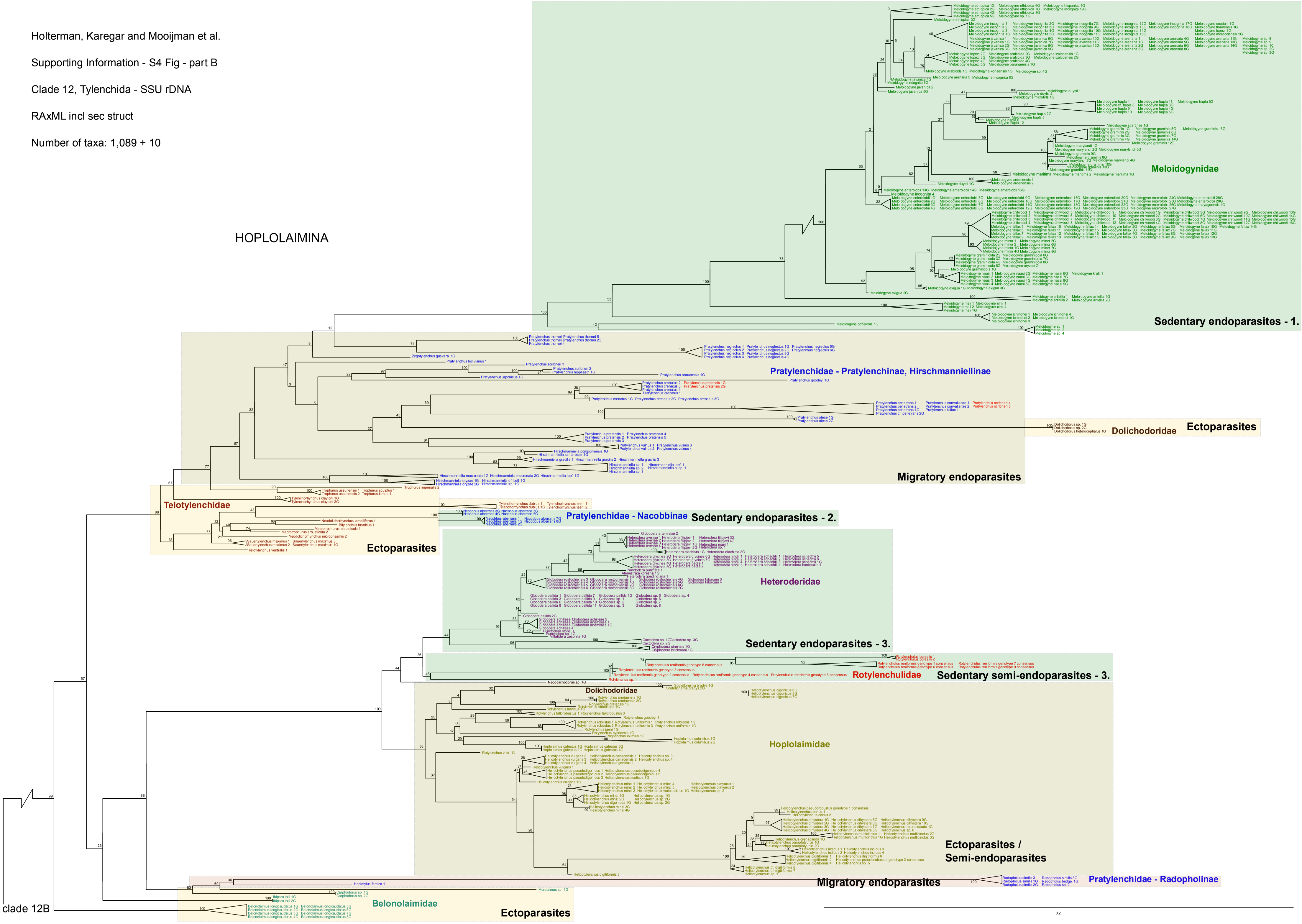

Disparate gain and loss patterns

Supporting information - S4 Fig. - part C

Clade 12, Tylenchida - SSU rDNA

Bayesian inference incl sec struct

10,000,000 generations

Number of taxa: 1,089 + 10 (S4 Fig)

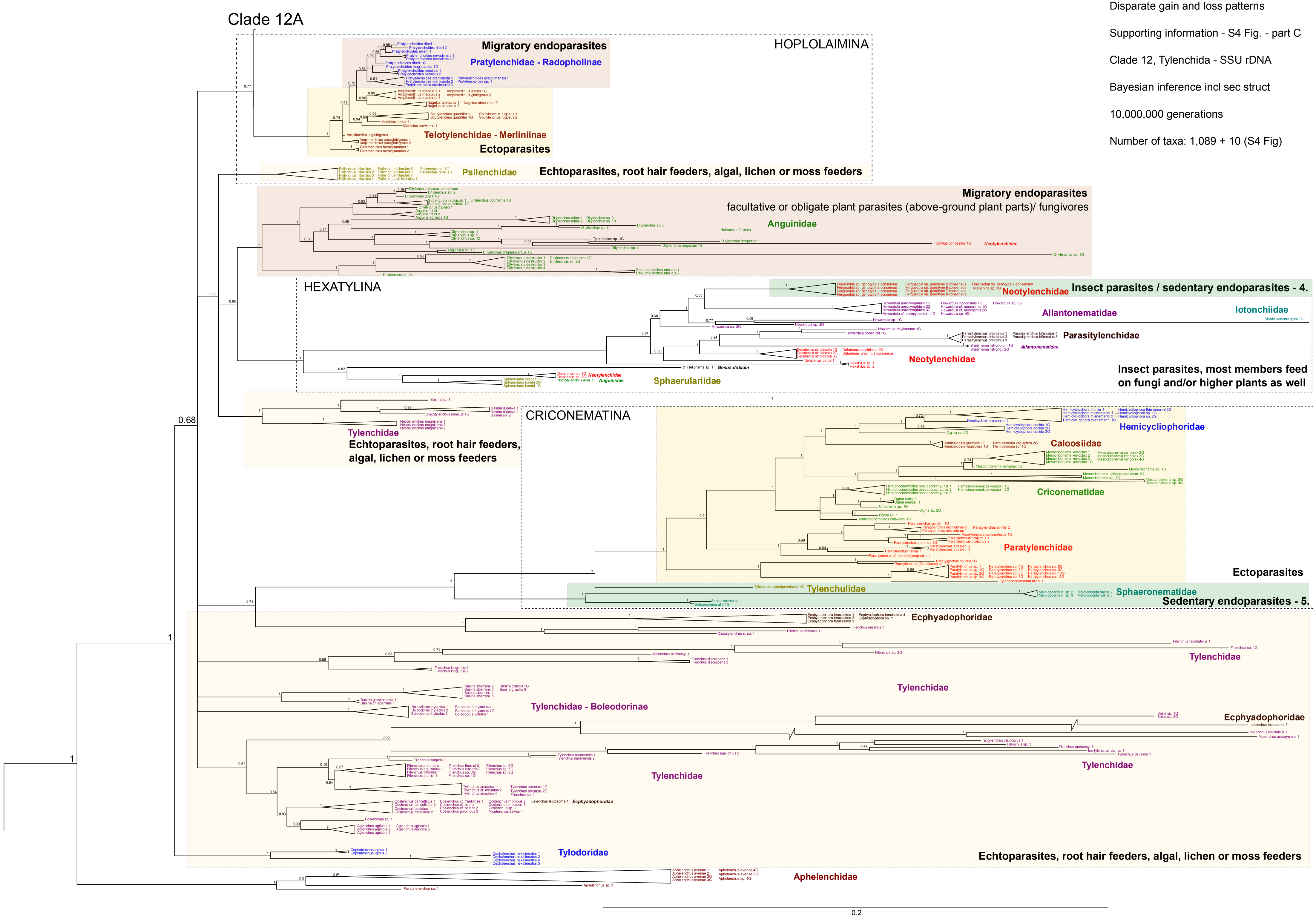

Disparate gain and loss patterns

Supporting information S4 Fig - part D

Clade 12, Tylenchida - SSU rDNA

RAxML incl sec struct

Number of taxa: 1,089 + 10 (Fig. 4A, B-2)

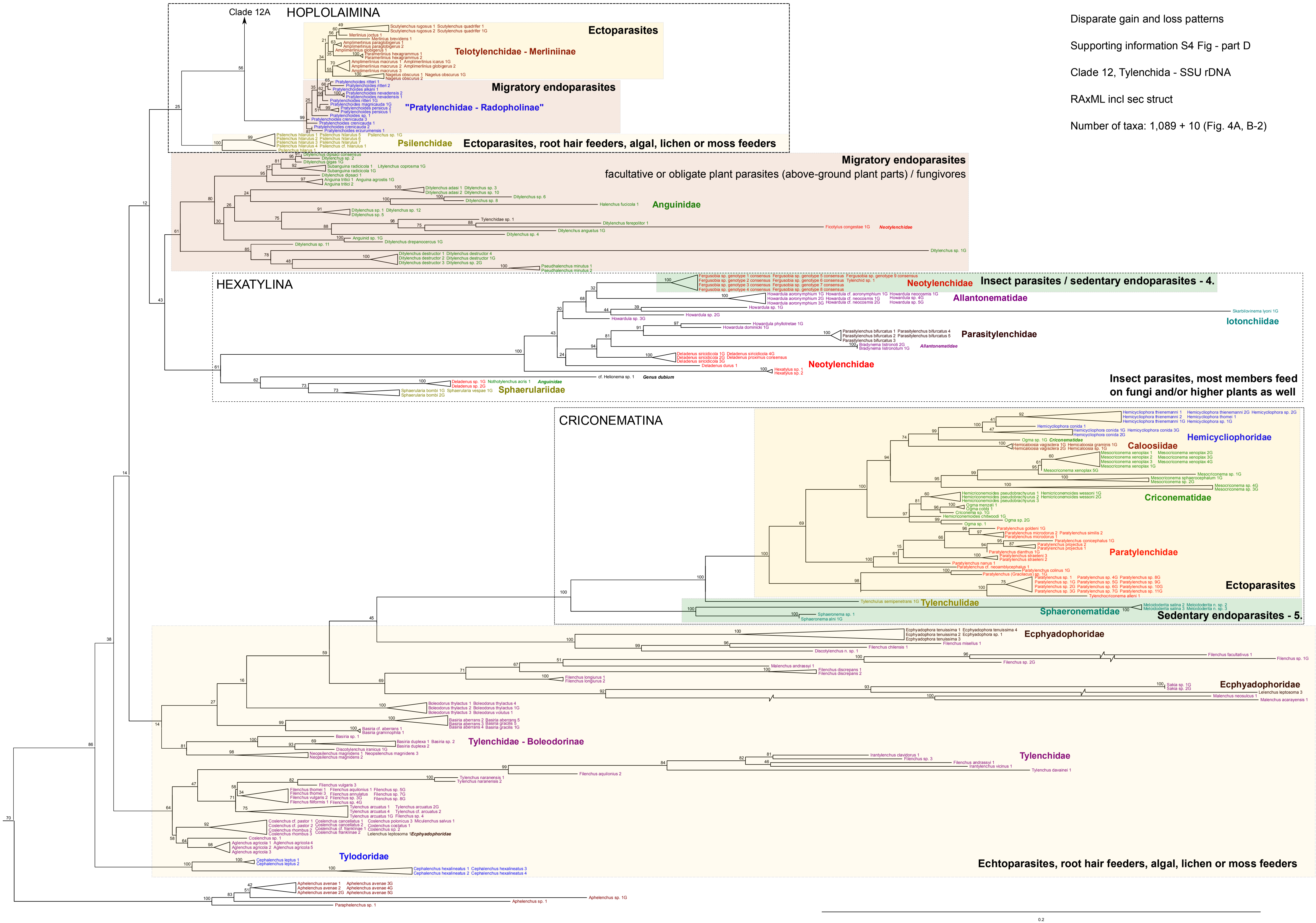

Supplement: S4 Fig — (PDF) [file pone.0185445.s004.pdf]
